# Factors Associated with Participation in Community Supported Agriculture (CSA) among Low-Income Households: A Scoping Review

**DOI:** 10.3390/nu16152450

**Published:** 2024-07-27

**Authors:** Karla L. Hanson, Claire Concepcion, Leah C. Volpe

**Affiliations:** Department of Public & Ecosystem Health, Cornell University, S2005 Schurman Hall, Ithaca, NY 14853, USA

**Keywords:** community supported agriculture, low-income households, cost offsets, community engagement, fresh fruit and vegetables, farm collaborations

## Abstract

Households with limited financial resources often struggle with inadequate access to healthy, affordable food. Community supported agriculture (CSA) has the potential to improve access to fresh fruits and vegetables, yet low-income households seldom participate due to cost and other barriers. Cost-offset (or subsidized) CSA reduces financial barriers, yet engagement varies widely among those who enroll. This scoping review explored factors associated with CSA participation among low-income households in the United States. Eighteen articles met the inclusion criteria, quantitative and qualitative data were extracted, the evidence was synthesized, and themes were developed. The findings suggested that women may be more likely than men to enroll in CSA. A lack of familiarity with CSA may hinder enrollment, whereas more education and self-efficacy for food preparation may facilitate participation. In terms of share contents, high-quality produce, a variety of items, more fruit, a choice of share contents, and a choice of share sizes may facilitate participation. In terms of CSA operations, a low price, good value, acceptance of Supplemental Nutrition Assistance Program (SNAP) benefits, close pick-up locations on existing travel routes, delivery of shares, clear communication, fostering a sense of belonging and trust, and educational support may support participation. Together these findings support 13 recommendations for cost-offset CSA implementation to engage low-income households.

## 1. Introduction

Fruits and vegetables (FVs) are known to have multiple health benefits [1] and are an important component of dietary recommendations [2]. However, adults and children in food insecure households and those with low household incomes consume fewer fruits and vegetables than their higher income counterparts who have more resources and greater food security [3,4,5,6,7,8,9]. Recent data suggest that only 6.8% of adults living in poverty meet vegetable intake recommendations [9]. Public health practitioners seek ways to improve access to affordable FVs to support health.

Community supported agriculture (CSA) is one approach to improving access to fresh, whole FVs. CSA brings local farms and households together, typically through an advance purchase of a share of the farm’s anticipated harvest [10]. However, low-income households seldom enroll in CSA due to up-front payments, cost, and other barriers [11,12]. Critics have stated that CSA reproduces social hierarchies and encourages exclusivity [10]. Yet, many farms are committed to making healthy food ‘more accessible to everyone’ [13]. Farms have attempted to make CSA more accessible to low-income households by subsidizing the price, eliminating up-front payments, and offering flexible payment plans. This model attempts to reduce some of the barriers to participating in CSA and is often referred to as cost-offset CSA.

Cost-offset CSA has been shown to improve food security and increase FV consumption among participants [11,14,15,16,17,18,19,20,21]. The extent to which participants engage in the cost-offset CSA may influence its impact. In a randomized controlled trial, the results revealed a dose–response relationship between the frequency of CSA pick-up and both adult FV intake and improved household food security. For example, for every additional week that households picked up their share, the daily FV intake increased by almost 1/8 cup among adults [20]. Additionally, households that picked up their share for 21 weeks (75th percentile) were 3.73 times as likely to be food secure as households that picked up for only 7 weeks (25th percentile) [20]. This finding is particularly important because levels of engagement in cost-offset CSA vary. For example, CSA share pick-up rates vary from 33% [22] to 86% [23] of weeks across studies and from 33–89% of weeks across locations within a single study [24]. Furthermore, reported drop-out rates can approach half before the end of the first season [17,22,25,26].

Cost-offset CSA has the potential to improve food security and nutrition, but low participation may jeopardize the potential impact of this nutrition intervention. A better understanding of the factors associated with CSA participation among low-income households is needed. To our knowledge, this is the first review article to synthesize the evidence of the factors that support and hinder participation in cost-offset CSA among low-income households. The goal of this research was to (1) conduct a scoping review to identify evidence of factors associated with participation in CSA among households with low incomes; (2) generate a list of emergent themes and aggregate their supporting evidence; and (3) draw upon these themes to make evidence-based practice recommendations for cost-offset CSA and to suggest areas for future research. These evidence-based recommendations may help to facilitate CSA participation by meeting the needs and preferences of low-income households (which may in turn support higher pick-up rates and reduced drop-out rates), thereby increasing the potential of CSA to improve food security and nutrition among residents of low-income households.

## 2. Methods

A scoping review methodology was used to examine the growing body of research on low-income households and CSA. The scoping review framework allowed us to summarize evidence regarding this relatively broad topic, synthesize findings generated by both qualitative and quantitative data and methods, and encompass both the scholarly and grey literature (e.g., reports and theses) [27]. Our methods were guided by the Preferred Reporting Items for Systematic reviews and Meta-Analyses extension for Scoping Reviews (PRISMA-ScR) guidelines [28].

### 2.1. Inclusion and Exclusion Criteria

We included studies that focused on participation in CSA among households with a low income in the United States (with or without a cost offset) and included at least one of three participation measures: (1) enrollment or the reasons for enrollment, (2) engagement such as picking up the share, and (3) indicators of satisfaction. We included only studies that reported the actual experiences or perspectives of adults in low-income households. One study also included the perspectives of children in low-income households, and these data were retained. Additional data from comparison samples (e.g., higher-income comparison households) were included and clearly marked. Evidence regarding outcome measures or CSA effectiveness did not address our aims and was not included. We excluded studies that did not meet these eligibility criteria, specifically, studies that reported on data from outside the United States, reported on measures beyond participation such as outcomes measures or CSA effectiveness, and studies that presented data from the perspective of someone other than a member of a low-income household (e.g., farmers and middle- or high-income CSA members).

### 2.2. Literature Search

The search strategy used Google Scholar and PubMed to identify journal articles, reports, and theses that used the following relevant search terms in the title: (“farm share” OR “food system” OR “food movement” OR “CSA” OR “CSAs” OR “community supported agriculture” OR “community-supported agriculture” OR “farm to family”) AND (“Income” OR “subsidized” OR “sociodemographic” OR “underresourced” OR “underprivileged” OR “SNAP” OR “food-insecure” OR “access”). Publication dates from 1 January 2000 through 28 July 2023 (the date of the last search) were included. Titles and abstracts were screened and advanced to the full-text review if they appeared to meet the inclusion criteria. The full-text review verified that the reference reported on data from the U.S., included a sample of low-income individuals or households, reported data from the perspective of adults with a low income, and included data on one or more of the three participation measures of interest (enrollment, engagement, and satisfaction). References cited by all articles that met the inclusion criteria after the full-text review were also screened for possible inclusion. If the title and/or abstract met the screening criteria, a full-text review of these additional articles was performed. Selected references that met the criteria above were added to the review.

### 2.3. Extraction

The following information was extracted from each eligible article: full citation, location/setting, study period, intervention design, study design for the reported measures of participation, sample size and type, and measures of participation assessed. We also extracted two types of evidence. First, descriptions of the characteristics and motivations of those who enrolled, the preferred share size and contents, and favorable farm operations and supports were extracted. These descriptions included both quantitative data (i.e., percentages reported or observed) and qualitative information (i.e., usual perceptions of interviewees or focus group participants). When present, extracted quantitative data were contextualized by data for the local setting (e.g., population rates). Second, we extracted data on associations between CSA enrollment, engagement or satisfaction and participant characteristics, share contents and frequency, or farm operations and supports. Associations were extracted only if they reported a bivariate test for association at a 95% confidence level or higher. All findings were extracted and placed into an Excel database.

### 2.4. Quality Assessment

Studies were evaluated according to level of design as follows: (1) randomized controlled trials (RCTs), (2) quasi-experiments with non-randomized comparison groups, and (3) observational, qualitative, and other non-experimental approaches. Opinions (level 4 studies) were not included in this review, nor was anecdotal evidence reported in the context of other study designs. Studies were also evaluated with respect to selection bias by noting (1) small samples (*n* < 30), (2) skewed sample selection (e.g., including the replacement of intervention drop-outs), (3) low response rates, and (4) multiple studies reporting on the same sample. In addition, we noted studies that reported data on perceptions of a *hypothetical* CSA among individuals largely unfamiliar with the CSA model and therefore particularly prone to bias.

### 2.5. Analysis

We conducted a descriptive analysis of the findings extracted from the selected articles. The first two authors separately reviewed each article and independently extracted the findings; any discrepancies in extraction were discussed until a consensus was reached. The third author grouped findings according to factors and by the measure of participation within factors, and then developed a list of initial themes. Initial themes were discussed amongst all three authors and themes were refined. Themes with three or more supporting studies were presented in tables and discussed below. Within each theme and participation measure, evidence was presented according to the sample type: first, interviewees responding to a description of a hypothetical CSA; second, low-income CSA members; and third, cost-offset CSA participants.

## 3. Results

### 3.1. Study Selection

**Figure 1:**
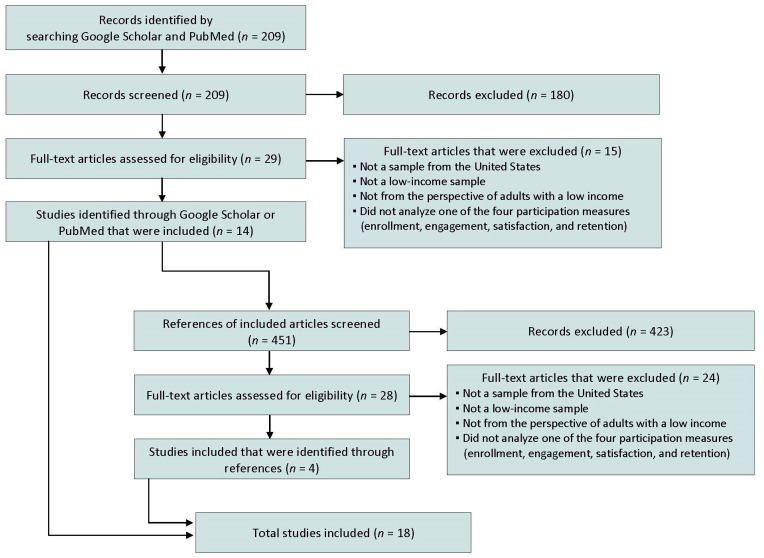
Selection of sources of evidence.

Two hundred nine studies were identified through the search strategy, all were screened, and 29 met the criteria for full-text review (see Figure 1). After the full-text review, 14 studies met all inclusion criteria and were retained [11,17,23,24,25,26,29,30,31,32,33,34,35,36]. The references of these fourteen sources were screened (*n* = 451), 28 met the criteria for the full-text review, and four met the inclusion criteria [16,22,37,38] and were retained, for a total of 18 sources (see Appendix A).

### 3.2. Study Characteristics

Multiple studies included samples from states in the Northeast [16,17,22,23,24,29,31,33,34,36,37] and west [23,24,25,26,30,31,33,34,36] regions of the United States, but only two from the Midwest [37,38] and none from the Southwest. North Carolina was the only state contributing samples from the Southeast, and multiple studies were conducted in that state [11,23,24,31,32,33,34,35,36]. Most studies (15 of 18) presented evidence collected since 2010 [16,17,22,23,24,25,26,29,30,31,32,33,34,35,36].

All findings extracted were observational or qualitative (level 3) in three broad categories: (1) surveys of low-income CSA members [16,30,37], (2) qualitative research that assessed interest in a hypothetical CSA [17,29,31,32,34], and (3) cost-offset CSA intervention studies [11,17,22,23,24,25,26,33,35,36,38]. Five studies provided a comparison group against which to compare CSA participants from low-income households: higher-income CSA members or those who paid the full price [30,37,38], cost-offset CSA applicants who did not enroll [16,23], or a comparison sample of low-income households that did not apply or enroll [16]. Five studies used qualitative methods with cost-offset CSA participants to assess satisfaction [11,22,25,33,36]. Four papers reported different analyses from one sample of participants in a cost-offset CSA intervention [23,24,33,36], but no duplicate findings were extracted.

Among the twelve studies that examined a cost-offset intervention, three interventions (six studies) provided price subsidies of 50% [16,22,23,24,33,36], three offered a 100% offset (free) [11,35,38], and three offered a subsidy somewhere in between [17,25,26]. Among interventions that were not free, average prices ranged from $5/week [17,22,25] to $13/week [23,24,33,36] and almost all accepted Supplemental Nutrition Assistance Program (SNAP) benefits as payment [17,22,23,24,25,26,33,36]. Most cost-offset CSA interventions also offered supports such as printed information and recipes [11,22,23,24,25,26,33,35,36] and/or FV education/preparation classes [17,23,24,25,26,33,35,36]. One cost-offset CSA intervention (four studies) provided kitchen tools [23,24,33,36] and one offered delivery [11].

### 3.3. Characteristics of CSA Participants from Low-Income Households

Five studies provided evidence that women may be more likely to enroll in cost-offset CSA (see Table 1). Two cost-offset CSA interventions used selection criteria that excluded men [32,35], but five others enrolled predominantly women (85–100%) without such restrictions [16,23,25,26,38]. One of these studies also reported that eligible mothers were more likely to enroll than eligible fathers (97.4% vs. 87.2%), despite both indicating interest in cost-offset CSA by completing a screening questionnaire [23].

Four studies suggested that college graduates may be more likely to enroll or engage in CSA. Three studies reported that low-income CSA members were more often college-educated than the state overall (82% vs. 31%) [30] and the county overall (62% vs. 28%) [38], and that cost-offset CSA participants were more often college-educated than a low-income comparison group (67% vs. 22%) [16], suggesting that a college education may facilitate enrollment. Another study reported that cost-offset CSA participants with a college education were more engaged; that is, they picked up shares a greater percentage of weeks than their counterparts with less education (82.6 vs. 57.8%) [24].

Three studies provided evidence that a lack of familiarity with CSA may hinder enrollment: most adults in low-income households were unfamiliar with CSA when it was described to them [29,31,32]. When a hypothetical CSA was described, some adults in low-income households perceived it as beneficial yet remained cautious about enrolling [31,32]. Three studies suggested that high self-efficacy for food preparation may support enrollment and engagement. In two studies, applicants to a cost-offset CSA had high self-efficacy for food preparation before the intervention began [16,25], and one other study reported that most cost-offset CSA participants knew how to prepare the FVs in their shares [11].

### 3.4. CSA Share Features Favorable to Low-Income Households

In general, CSA produce was perceived as high quality, which may facilitate enrollment and satisfaction. Ten studies provided evidence that adults in low-income households perceive FVs from CSA farms to be of high quality, whether or not they ever participated in CSA (see Table 2) [11,17,22,25,26,30,31,34,35,37]. Two studies reported that adults would be motivated to enroll in a hypothetical CSA because the FVs were high quality [31,34], one study reported that FV quality motivated low-income CSA members to enroll [37], and seven studies reported that low-income CSA members and cost-offset CSA participants [11,17,22,25,26,30,35] were satisfied with the quality of produce. Adults described FVs from CSA as fresh [11,17,22,25,31], better tasting [11,35], and free of pesticides [22,37].

Six studies provided evidence that suggested CSA shares that include a variety of FVs may support enrollment and satisfaction. Two studies reported that adults and children in low-income households want a variety of FVs in a hypothetical CSA [31,34]. Four studies reported that cost-offset CSA participants were satisfied with the variety of FVs they received [11,22,35,36], and appreciated receiving FVs they perceived as too expensive for them to purchase at the grocery store [22,35]. One study also reported that some cost-offset CSA participants expressed frustration that their shares lacked variety [36]. The most commonly preferred FVs were green beans, lettuce, and tomatoes [11,31], as well as broccoli, carrots, corn, onions, peas, peppers, and potatoes [31]. In one study, more than half of adults from low-income households requested broccoli, carrots, cucumbers, green beans, peppers, potatoes, and tomatoes in a hypothetical CSA share [31]. Three studies also found that cost-offset CSA participants wanted more fruit in their share [11,17,22].

Three studies reported mixed reactions to unfamiliar produce items: some cost-offset CSA participants enjoyed the challenge of using new FVs [22,36] and others left unfamiliar items uneaten [11,36].

Four studies provided that a choice of FVs in CSA shares may support enrollment and satisfaction. When interviewed about a hypothetical CSA, adults from low-income households described wanting to choose the FVs in their share [31,32], and indicated that not being able to choose would be a barrier to enrollment [29,31,32]. Cost-offset CSA participants were more satisfied when they were able to choose their own FVs and perceived the share to have greater value when they selected the FVs, and those who were not offered self-selection requested it [36].

Eight studies provided evidence that a choice of share sizes may support engagement and satisfaction by meeting diverse household needs. Six studies reported that cost-offset CSA participants received an adequate or more than adequate quantity of FVs in their CSA shares [11,17,23,25,26,36]. Three studies reported that some cost-offset CSA participants thought the share was too small [11,17,22]. In three cost-offset CSA interventions (six studies), participants were offered multiple share sizes [11,22,23,24,33,36]. In one study, participants were offered two shares sizes and requested a third size because the full share was too large but the half share was too small [22]. When participants were offered a choice of share sizes, they picked up more shares than when it was not offered (76.8% vs. 57.7% of weeks) [24].

### 3.5. Cost-Offset CSA Operational Practices Favorable to Low-Income Households

Eight studies provided evidence that a low price may motivate enrollment in cost-offset CSA [17,30,31,32,34,36,37,38] (see Table 3). When interviewed about a hypothetical CSA, more than half of adults in low-income households reported that a low price was important for enrollment [31], and some perceived that they would be unlikely to join due to the high cost [32,34]. As the price increased, interest in CSA seemed to wane. One study reported that 67% of adults were interested in the hypothetical CSA at a reduced price but only 18% were interested at full price [17]. Among CSA members from low-income households, affordability was a more important motivation to join than among members from higher-income households [30]. Similarly, among cost-offset participants, receiving low-cost produce was what attracted many participants in the first place [36]. Five studies reported that cost-offset CSA participants were satisfied with the CSA’s ‘value’ (generally described as price and quality) [11,17,22,30,36].

Three studies suggested that acceptance of SNAP benefits may support CSA enrollment and engagement. When asked about a hypothetical CSA, the majority of adults from low-income households were interested in paying with SNAP benefits [17,29,31]. In one study, most cost-offset CSA participants did use their SNAP benefits to cover payments for their CSA shares [17].

Seven studies provided evidence that CSA participants from low-income households desired convenient pick-up locations [17,30,31,33,34,35,36], which they defined as nearby or on existing travel routes. When asked about a hypothetical CSA, 66% of adults from low-income households reported that a convenient pick-up location would be a facilitator to enrollment [31], and most were willing to travel 15 min or less to get their CSA shares [34]. CSA members from low-income households placed greater importance on a nearby pick-up location than their higher-income counterparts [30]. Similarly, cost-offset CSA participants wanted pick-up locations near home [36] or near daily activities like transporting children [33], and faced challenges with pick-up locations that were further away [35] or were an ‘extra errand’ [36]. One study reported that pick-up rates were higher among households whose children remained enrolled in Head Start (the pick-up location) than among those whose children withdrew and therefore they had to make a special trip to pick-up the CSA share (81% vs. 57%) [17].

Three studies provided evidence that delivery of CSA shares was desired by adults in low-income households. When asked about a hypothetical CSA, many adults requested that their CSA shares be delivered in order to save scarce time [31]. When offered delivery, most cost-offset CSA participants selected it (68%) [11], and among participants not offered delivery, some requested it [33].

Seven studies reported mixed results regarding organization, communication, and a sense of belonging among CSA participants from low-income households. Two studies reported that some cost-offset CSA participants were frustrated by disorganization, such as poorly organized pick-up locations [22,36], confusion regarding share contents [36], and payments systems that seemed confusing or inaccurate [22,36]. CSA members from low-income households valued ease of communication with the farmers more than their higher-income counterparts [30]. Cost-offset CSA participants also reported that they were satisfied with clear labelling at pick-up locations where FVs were self-selected [36], and appreciated instrumental supports like reminders [25,36] and newsletters [36]. Three studies described positively the context or environment in which the CSA operated. Most cost-offset CSA participants rated the environment as ‘excellent’ (85%) [26], and described a sense of ‘belonging’ [25], support from other cost-offset CSA participants [36], and farmers who ‘trusted them,’ and were ‘accommodating’ [36]. In contrast, CSA members from low-income households were somewhat less likely to feel a part of the CSA community than their higher-income counterparts (46% vs. 63%) [38]. Geospatial data showed that cost-offset CSA participants who travelled to pick-up locations geographically further from their homes also spanned a greater socio-economic distance from their own neighborhoods [33].

Five studies provided evidence that educational supports may facilitate enrollment and satisfaction with cost-offset CSA. When asked about a hypothetical CSA, one study reported that adults from low-income households described needing both ‘new and healthy recipes’, as well as ‘hands-on’ cooking instruction [29]. Three studies reported that cost-offset CSA participants found printed materials like recipes and newsletters [22,26], active demonstrations [26], and informal advice from the farmers [36] all to be helpful educational supports. Some cost-offset CSA participants wanted more, including how to store highly perishable FVs [11,36] and how to preserve FVs with canning and freezing [22,36].

## 4. Discussion

The descriptive analysis identified 21 themes supported by correlational evidence from three or more studies of participation in CSA among low-income households. Together these findings support 13 recommendations for evidence-based practices that may support enrollment and engagement in cost-offset CSA by low-income households. We also identified five areas for future research.

### 4.1. Evidence-Based Recommendation for Cost-Offset CSA

1. Focus on recruiting women: CSA participants from low-income households (with or without a cost offset) were predominantly women (85–100%) [16,23,25,26,38]. This is consistent with other data that suggest women assume the primary responsibility for meal planning, preparation, and shopping [39,40,41]. Given this gendered context, recruitment efforts that focus on recruiting women into cost-offset CSA may be most successful.
2. Build awareness around CSA: Most adults in low-income households were unfamiliar with CSA (71–87%) [31,32] which may hinder enrollment. Teaching people about the CSA model may be an important part of recruiting new members from low-income households but may be too time consuming for farms to integrate into daily public interactions. Little is known about how best to educate the public about how CSA works. Galt et al. (2017) reported that when deciding whether to enroll in CSA, low-income households were more likely than higher-income households to use websites (21 vs. 12%) and social media (16 vs. 4%) as important sources of information [30]. This suggests that online resources to address the lack of familiarity with the CSA model may be effective and time saving.
3. Amplify message that CSA produce is high-quality: Ten of eighteen reviewed studies suggest that there is a widespread belief that CSA produce is high quality among both CSA participants and adults with no CSA experience [11,17,22,25,26,30,31,34,35,37]. Fresh, quality, and organic FVs were motivators to join a CSA [31,34,37], and fresh, quality, organic FVs and taste were important to satisfaction among CSA members [11,17,22,25,26,30,35]. However, it is important to note that two studies reported dissatisfaction with the quality of produce [11,36], including the presence of bugs, mold, and quick spoilage [36]. Recruitment efforts could amplify positive perceptions of CSA quality while also being transparent about the variable aesthetics of farm produce [42] relative to homogenous grocery store produce [43] and creating realistic expectations.
4. Include a variety of FVs and/or 5. Offer self-selection of FVs: The reviewed studies suggest that a variety of FVs and more fruit are desired in CSA shares. Three items were reported in multiple studies as preferred by adults and children in low-income households: potatoes (43–51%), carrots (50–75%), and tomatoes (35–60%) [31]. Three studies described cost-offset CSA participants as receiving less fruit than they expected or desiring more fruit than they received [11,17,22]. Food preferences vary and may be influenced by social and cultural norms, making it challenging to select FVs enjoyed by all [44,45]. Eight of the reviewed studies had predominantly white, non-Hispanic samples [16,23,24,25,30,31,34,36], and race and ethnicity were not reported in six other studies [11,32,33,35,37,38], providing little information about participants’ cultural contexts. Farms often choose what goes into CSA shares, but sometimes they offer members choice about share contents [46]. The reviewed studies support the idea that the self-selection of share contents (sometimes called a ‘market style’ CSA) may facilitate enrollment and satisfaction [29,31,32,36]. If this is not possible, offering shares that have variety [11,22,31,34,35,36], including some specialty items that are perceived as otherwise unaffordable [22,35], and adding more fruit [11,17,22] may also support CSA participation by low-income households.
6. Offer multiple share sizes: We found ample evidence that FV quantity in CSA shares was usually adequate [11,17,23,25,26,36]. Cost-offset CSA participants described the size as ‘just right’ and 82–93% reported using all the FVs most weeks [17,23]. There was some evidence that the quantity was insufficient relative to the expectations or family size [11,17,22], and limited evidence that shares were too large. Cost-offset CSA participants frequently had a choice of two share sizes, and some wanted a third ‘in-between’ size. Moreover, importantly, when cost-offset CSA participants were offered a choice of share sizes, they picked up their shares on more weeks (77 vs. 58%) [24]. Farms can support CSA enrollment and engagement by offering multiple share sizes to meet diverse needs.
7. Keep prices low: The reviewed studies provide ample evidence that low prices, affordability, and ‘good value’ are important to low-income households. Four studies reported that a low cost or affordability would be motivators to join a hypothetical CSA [17,31,32,34], and four more reported they did motivate participation among CSA members [30,36,37,38]. Five studies described satisfaction with the ‘value’ of CSA shares [11,17,22,30,36], and two quantified this satisfaction as nearly universal (91–93% of CSA members) [11,17]. However, meeting general value expectations may be challenging for farms. The price of CSA varies based on factors such as size and duration. In the Northeast (for example), the CSA season typically runs for 22 weeks with an average cost of $400–$500 for a standard share (or about $20/week) [47]. Two prior studies suggests that low-income consumers are willing to pay approximately $10/week for a small CSA share and $15/week for large share [34,35]. All reviewed studies that tested a cost-offset CSA intervention included a price reduction of 50% or more. The estimated willingness to pay $10–15/week supports the idea that cost offsets of at least 25% (and likely 50%) may be needed for low-income households to participate in a CSA (given average price of $20/week). The cost offsets or subsidizes may be obtained through member donations, fundraising, or grants [13]. Community organizations may be able to ease the burden on farms by securing and providing the subsidies needed to keep the price within this threshold [48,49].
8. Accept SNAP benefits: Most cost-offset CSA programs accepted SNAP as weekly payment for CSA shares, and potential participants wanted this option (51–59%) [16,17,29]; many cost-offset CSA members used SNAP as payment (67%) [17]. This is particularly important given that many low-income households participate in SNAP. Data from the USDA show that over 80% of people eligible for SNAP participate in the program [50]. Farmers’ markets are a common location for CSA pick-up [51]; however, less than half of the nearly 7000 farmers’ markets are authorized to accept SNAP benefits [52]. In order to serve low-income households, farms may need to become authorized to accept SNAP payments directly or work with a community partner who can process SNAP payments [49].
9. Offer convenient pick-up locations and/or 10. Provide delivery: We found substantial evidence that CSA members desired convenient CSA pick-up. Low-income households preferred pick-up locations that were close to home [30,34,35,36] and/or near places that participants were already travelling (e.g., school and childcare) [17,31,33,36]. One study reported that a short distance to pick-up was more important to CSA members from low-income households than it was for their higher-income counterparts [30]. Three studies reported that delivery was requested by cost-offset CSA participants and potential participants [11,31,33], and that participants used the service when offered [11,33]. Constraints on time and physical mobility may contribute to a need for convenience. Low-income households are time poor, as well as have limited financial resources [53]. Additionally, nearly one-quarter of disabled people live in poverty [54]. Participation by low-income households may be supported by locating pick-up sites close to affordable housing and/or services such as health centers and Head Start programs and partnering with delivery services. Distribution can be time-consuming for farms, and in response to this, community groups and neighborhood volunteers have sometimes stepped in to assist with CSA distribution and delivery [49].
11. Prioritize clear communication: CSA participants from low-income households valued clear communication, including labelling of FVs, newsletters, and reminders, and were frustrated by disorganized pick-up and payment systems. CSA members from households with a low income placed greater importance on ease of communication with CSA staff/farmer than their higher-income counterparts [30]. CSA often uses a subscription model that requires up-front payment [46], whereas cost-offset CSA eliminates up-front payments and offers flexible payment plans. This requires tracking and managing multiple payments, which can intensify the need for clear and consistent communication, particularly regarding payment. Clear communication systems may be particularly important to retain CSA members from low-income households who need to make multiple payments.
12. Foster an environment of belonging and trust: Some CSA participants from low-income households described a supportive and trusting environment that sometimes included socializing and instrumental support [25,26,36]. However, cost-offset CSA participants were somewhat less likely to feel a part of the CSA community compared to their higher-income counterparts [38]. One study reported that cost-offset CSA participants who travel further to pick-up locations enter starkly different socio-demographic contexts [33]. Individuals with a low income often struggle with inadequate social networks and support [55,56]. A positive environment that supports trust, belonging, and socialization may facilitate participation in cost-offset CSA.
13. Provide educational support: Cost-offset CSA participants appreciated educational support and found it helpful [11,22,26,36]. Three studies suggested specific interest in food storage and preservation [11,23,36]. However, Garner et al. (2021) reported very low attendance when education classes were offered as a separate activity in a cost-offset CSA program [23]. Thus, integrating education into CSA pick-up may be a more feasible mode of education for busy households. When cooking demonstrations and tastings were offered at pick-up, 71% of cost-offset CSA participants rated this component highly [26].

### 4.2. Opportunities for Future Research

1. Investigate why adults with less education do not enroll in CSA: Many low-income CSA participants were college graduates (62–82%) [16,30,38], which is atypical given that only 13% of the low-income adults have a college degree [57]. Future research should investigate why women with less education do not enroll in cost-offset CSA and identify potential adaptations to better meet their needs.
2. Investigate ways to develop self-efficacy for food preparation: CSA participants from low-income households had high self-efficacy for food preparation (even before CSA enrollment) [11,16,25]. Future research should explore mechanisms by which self-efficacy for food preparation can be developed prior to or in the context of cost-offset CSA participation.
3. Investigate which FV varieties are both preferred by households and profitable for farms: Crop planning is an important component of developing a successful CSA. The profitability of different crops can vary depending on factors such as the labor intensity of growing and harvesting, field space or acreage needed, and the time needed for crop maturation [58]. In addition, little is known about the specific items that CSA members from low-income households prefer, and taste preferences may be highly dependent on local cultures and ethnicities [44,45]. Future research should investigate methods to identify FVs that are preferred by low-income households and also considered profitable for farms within a local area.
4. Investigate the association between offering a CSA with FV self-selection and participation: Self-selection of FVs within the CSA share was popular among CSA members from low-income households [29,31,32,36]. To our knowledge, no data are available on how common self-selection of FVs is in CSA operations. Future research should document the prevalence of self-selection mechanisms and test the association with CSA participation among low-income households.
5. Investigate CSA engagement at different prices: Among the cost-offset CSA interventions reviewed, subsidies varied from 50% to 100% (free) [11,16,17,22,23,24,25,26,33,35,36,38], and prices among shares that were not free varied from $5 to $13/week [11,17,22,23,24,25,26,33,35,36]. However, participation at different subsidies and price levels was not compared in any study. Future research should compare CSA participation by low-income households at different prices and cost-offset levels.

### 4.3. Importance of Community Engagement

In a national survey, nearly five hundred CSA managers were asked about their interest in cost-offset programs for low-income households and over two-thirds reported being very interested or possibly interested in this mechanism [49]. Farms with experience implementing a cost-offset CSA report that they believe it is a worthwhile addition to their business model [59]; however, farms that were exploring this mechanism cited additional management and administrative duties as barriers to implementation [13,60].

The recommendations made in this paper have the potential to enhance participation in cost-offset CSA among low-income households, but required changes in finances and operations may mean that some farms struggle to implement a cost-offset CSA program alone. A community-engaged approach to CSA has been advocated for in other studies and might benefit from enacting these recommendations as well [61]. For example, some of the recruitment strategies that we present here may be best led by a community organization or community liaison who already has existing relationships with adults in low-income households and can facilitate building trust with farms [13,48,49]. Furthermore, community organizations have relieved some of the pressure on farms by helping with distribution and delivery, managing flexible payment structures, processing SNAP payments, financing subsidies to keep prices low, and delivering education [48,49].

### 4.4. Limitations

There are several limitations of this review that deserve to be noted. First, five studies used small samples (*n* < 30) [11,17,25,29,35], which may not be representative of the population and limit the generalizability of findings. Second, five studies collected data on interest in or willingness to pay for a hypothetical CSA [17,29,31,32,34]. Because many adults in low-income households were completely unfamiliar with CSA [29,31,32], the validity of these results may be limited. Third, four studies used sampling techniques that introduced potential bias. Three of these studies replaced CSA participants who dropped out [11,25,26] (two had dropout rates approaching half [25,26]), which may bias estimates of engagement upwards. Another study conducted post-intervention focus groups to which they only invited the participants who had picked-up the most CSA shares [22]. Finally, four studies reported different data but from the same sample of participants in a cost-offset CSA [23,24,33,36], which therefore over-represents the perspectives of these participants.

## 5. Conclusions

This review suggests evidence-based recommendations to support more robust enrollment and engagement in cost-offset CSA by low-income households. For recruitment into CSA, low-income households need to be educated about how CSA works and amplify perceptions of high quality FVs. Farms may need to offer low-income households a choice of shares sizes to meet diverse needs, as well as a variety of FVs or the option to self-select share contents. Cost-offsets of at least 25% (and likely 50%) are needed to achieve CSA prices of $10–15/week (which low-income households viewed as affordable), with the option to pay with SNAP benefits. Flexible systems for payment, pick-up (or delivery), clear communication, and a supportive environment all may be needed for CSA to be broadly feasible for and acceptable to low-income households. These recommendations may be challenging for farms, and a community-engaged approach may support their implementation.

## Figures and Tables

**Table 1:** Characteristics of Community Supported Agriculture (CSA) participants from low-income households.

Theme	Participation	Findings	Action
Women appear more likely to enroll	Enrollment	A total of 97% of cost-offset CSA participants were women [23].	Focus on recruiting women
		In total, 96% of applicants to a cost-offset CSA were women [16].	
		In total, 85% of cost-offset CSA participants were women [38].	
		A total of 100% of cost-offset CSA participants were women [25].	
		In total, 92% of cost-offset CSA participants were women [26].	
		Eligible mothers were more likely to enroll in the cost-offset CSA than eligible fathers (97.4% vs. 87.2%) [23].	
Adults with more education appear more likely to enroll and engage	Enrollment	CSA members from households with a low income had more education than adults in households statewide (82% vs. 31% had a college degree) [30].	Investigate why adults with less education do not enroll in CSA
		Cost-offset CSA applicants had more education than a comparison sample of caregivers in low-income households (67% vs. 22% had graduated from college) [16].	
		A total of 62% of cost-offset CSA participants held college degrees, which is fewer than CSA participants who pay the full price (82%) but greater than the county overall (28%) [38].	
	Engage-ment	Cost-offset CSA participants with a college education (82.6 vs. 57.8%) picked up CSA shares a greater percentage of weeks than their counterparts with less education [24].	
Lack of CSA familiarity may hinder enrollment	Enrollment	Few focus group respondents were aware of CSA when a hypothetical CSA was described, and none had been a member before [29].	Build awareness about CSA
		Most interviewees (71%) were unfamiliar with CSA. When the hypothetical CSA was described, they could articulate its benefits but most remained uncertain about enrolling [31].	
		Few interviewees were familiar with CSA (13%) and less than half reported being interested in enrolling when the planned cost-offset CSA was described to them (47%) [32].	
High self-efficacy for food preparation may promote participation	Enrollment	Cost-offset CSA applicants had high self-efficacy for cooking and meal preparation (median of 4.2 on 5-point scale) [16].	Investigate ways to develop self-efficacy for food preparation
		Self-efficacy to prepare and to consume FVs were high (4.5 and 4.1 on a 5-point scale) among cost-offset CSA participants pre-intervention [25].	
	Engage-ment	Most cost-offset CSA participants knew how to prepare the FVs included in the CSA share [11].	

**Table 2:** Community Supported Agriculture (CSA) features favorable to low-income households.

Theme	Participation	Findings	Action
High-quality produce may facilitate enrollment and satisfaction	Enrollment	More than half of interviewees mentioned the freshness of produce as a facilitator to hypothetical CSA participation (54%) [31].	Amplify the message that CSA produce is high quality during recruitment
		Some interviewees were willing to enroll in the hypothetical CSA no matter the price relative to the supermarket because of perceived quality: ‘*But you may have better quality. And that’s my thing, if I know it is better quality, I wouldn’t mind paying that price*.’ [34].	
		Food free of pesticides was an important motivator for CSA members with a low income [37].	
		Food that was not genetically engineered was an important motivator for CSA members with a low income [37].	
		Knowing where/how food is grown was an important motivator for CSA members with a low income [37].	
	Satisfaction	Low-income CSA members reported that high-quality produce was important (4.9 on a 5-point scale) [30].	
		Low-income CSA members reported that the farm’s agricultural practices (e.g., organic) were important (4.6 on a 5-point scale) [30].	
		Almost all cost-offset CSA participants agreed that the fruit and vegetables (FVs) from the share tasted better because they were freshly picked: I am “*amazed at how much better fresh green beans were than canned ones*.” [11].	
		Cost-offset CSA participants thought that the vegetables were fresher than what they purchased from the supermarket [22].	
		Cost-offset CSA participants appreciated that their produce came from local farms with minimal pesticides and did not mind the dirt because it had a “natural feel.” [22].	
		All the cost-offset CSA participants agreed or strongly agreed that the FVs were fresh and high quality [17].	
		Cost-offset CSA participants indicated that the cost offset provided them access to a wide variety of fresh and/or organic vegetables. “*When you’re poor … I can’t afford to go and buy a vegetable and have it taste bad. And waste my money. I don’t have the money to do that. This gave me the opportunity to taste things*.” [25].	
		Most cost-offset CSA participants rated the quality of FVs as excellent (88%) [26].	
		Cost-offset CSA participants reported enjoying the better flavor of local produce compared with grocery store produce [35].	
A variety of FVs are enjoyed by adults and children and desired in CSA shares	Enrollment	Adults and their children preferred 64 different FVs (44 of which were locally grown in their state), which suggests a wide range of preferences across families and individuals interviewed about a hypothetical CSA [31].	Include a variety of FVs
		Most interviewees preferred green beans (75%), corn (63%), tomatoes (60%), broccoli (55%), onions (53%), and carrots (50%), and many also liked lettuce (48%), peppers (45%), potatoes (43%), and peas (38%) [31].	
		Most interviewed children preferred carrots (75%) and broccoli (70%), and many liked tomatoes (35%), corn (30%), peas (30%), and peppers (30%) [31].	
		More than half of interviewees requested broccoli (78%), cucumbers (73%), green beans (61%), carrots (61%), peppers (58%), tomatoes (51%), and potatoes (51%) in their hypothetical CSA share [31].	
		Interviewees mentioned that they preferred having variety in the hypothetical CSA share: ‘*I just like a variety of different stuff. And the children, they hate to eat the same things all over and over and over again*.’ [34].	
	Satisfaction	Cost-offset CSA participants enjoyed receiving different fruits and vegetables [22].	
		Cost-offset CSA participants reported enjoying the variety of FVs and the chance to expose their children to new items [35].	
		Tomatoes, beans, and salad greens were the favorite vegetables among cost-offset CSA participants [11].	
		Cost-offset CSA participants reported enjoying the chance to eat foods that were too expensive to purchase at the store [35].	
		Some cost-offset CSA participants expressed frustration that shares had ‘*a lot of one thing and a little bit of something else*’ [36].	
More fruit in shares was desired	Satisfaction	A minority of cost-offset CSA participants reported that they received less fruit than expected [11].	
		Most cost-offset CSA participants were happy with the types of FVs received (79%), but some wanted more fruit (43%) [17].	
		Cost-offset CSA participants wanted more fruit and herbs in the shares [22].	
Mixed reactions to unfamiliar produce	Satisfaction	Cost-offset CSA participants appreciated exposure to new vegetables through the CSA [22].	Investigate which FVs are preferred
		Cost-offset CSA participants commented on unfamiliar FVs: a ‘s*melly green thing*’ (basil), ‘*red round things*’ (radishes), and Swiss chard [11].	
		Cost-offset CSA participants described receiving FVs that they could not identify or name: *“Lot of times it would just go bad and I would add them to my compost area and I’m like ‘Sorry, I don’t know what to do with you. You’re cute but I have no idea what to do with you.’”* [36].	
		All cost-offset CSA participants described being motivated to use any unfamiliar FVs to justify the money they had spent [36].	
		Some cost-offset CSA participants described how children enjoyed new FVs: *“Anytime I would bring the CSA home [to my kids] and … they would just be like looking through it, ‘What did we get this week? What did we get?” […] It was just really fun to watch.”* [36].	
A choice of share contents was preferred	Enrollment	The primary barrier to participation in the hypothetical CSA was the unwillingness to prepay for a box of FVs with limited food dollars without knowing exactly what would be in it: ‘*So that means that what would be in the box, if I didn’t want it, it’s in the box anyway*…’ [29].	Offer self-selection of FVs and investigate the association with CSA participation
		In total, 44% of interviewees described the FV choice as important to enroll in a hypothetical CSA, and 46% described a lack of choice as a barrier [31].	
		Interviewees described a choice of FVs as important and a lack of choice as a barrier to enrolling in a hypothetical CSA. “*I don’t like anybody picking products for me. I like to pick my own*.” [32].	
	Satisfaction	Cost-offset CSA participants seemed more satisfied when they chose their own FVs, and those without a choice requested it: ‘*Everybody agrees, there should be some sort of option like if there’s something you’re definitely not going to eat […] it’s kind of wasteful.*’ [36].	
		Cost-offset CSA participants perceived a lack of FV choice to decrease the value: “*When I go to the grocery store and I spend ten dollars, I’m buying what I know I like, rather than just spending whatever that’s costing, and half of it, I don’t know what it is and I’m not gonna eat it.*” [36].	
A choice of share sizes was desired	Engagement	Most cost-offset CSA farms offered multiple share sizes (69.2%), and participants picked-up more shares when this was offered than when it was not (76.8% vs. 57.7% of weeks) [24].	Offer multiple share sizes
	Satisfaction	Cost-offset CSA participants wanted three share sizes because the full share was too large but the half share was too small [22].	
		A few cost-offset CSA participants decreased their share size: “*I didn’t know how much stuff is actually in a share. You know, it said so many units, but here I’m thinking okay we’re going to get maybe three apples. […] I didn’t think those boxes were going to be packed to the max.*” [36].	
The quantity of FVs was adequate	Engagement	Most cost-offset CSA participants reported using all or most of the cost-offset CSA produce throughout the season (82%) [23].	
	Satisfaction	Almost half of cost-offset CSA participants agreed/strongly agreed that CSA shares always had more FVs than they could eat (45%). [11].	
		Most cost-offset CSA participants were pleased with the quantity of FVs most weeks (79%) and used them all most weeks (93%). [17].	
		All cost-offset CSA participants reported that the amount of vegetables was ‘just right’ [25].	
		Most cost-offset CSA participants reported that the quantity of vegetables was just right (71%) [26].	
		Cost-offset CSA participants reported that the share size was adequate or more than adequate, and no one wanted to increase their size [36].	
The quantity of FVs was insufficient	Satisfaction	A minority of cost-offset CSA participants had large families who easily ate all the FVs in the share (23%) [11].	
		Many cost-offset CSA participants would have liked even more FVs (71%) [17].	
		Cost-offset CSA participants were disappointed with the limited quantity and variety at the beginning of the season [22].	

**Table 3:** Community Supported Agriculture (CSA) operational practices favorable to low-income households.

Theme	Participation	Finding	Action
A low price may motivate enrollment	Enrollment	In total, 54% of interviewees mentioned a low cost as important for enrollment in a hypothetical CSA [31].	Keep prices low and investigate CSA engagement at different prices
		Interviewees described it as unlikely they would be able to join a hypothetical CSA due to the perceived high cost [32].	
		Interviewees perceived the price of a hypothetical CSA as higher than the supermarket. “*If it was the cheaper price, I would buy from the supermarket. I would have to go where I could get the most of my money, better bang for your bucks.*” [34].	
		A total of 67% of interviewees were interested in a hypothetical CSA if it was offered at a reduced price, but only 18% indicated that they would still be interested even if they had to pay full price [17].	
		Low-income CSA members rated ‘saving money’ as a more important reason to join and ‘affordability’ as a more important CSA attribute than their higher-income counterparts [38].	
		CSA members from households with a low income were more motivated to enroll in CSA by ‘saving money’ than their higher-income counterparts (3.4 vs. 2.8 on 10-point scale) [30].	
		Affordable food is important to CSA members with a low income. As income increases, this motivation drops [37].	
		Receiving low-cost produce was what attracted many cost-offset CSA participants [36].	
CSA was perceived as a good value or helped save money	Satisfaction	CSA members from low-income households reported ‘affordability’ as a more important CSA attribute than their higher-income counterparts (4.3 vs. 3.8 on a 5-point scale) [30].	
		A total of 93% of cost-offset CSA participants thought the cost of the fruit and vegetables (FVs) was a good value [17].	
		Cost-offset CSA participants described how spending more money for higher quality FVs was acceptable [36].	
		A total of 91% of cost-offset CSA participants reported that they spent less money because they did not buy as much produce at the store [11].	
		Most cost-offset CSA participants reported the price was affordable and they saved money compared to grocery store prices. “*I thought that the cost was very reasonable. […] We were making more food than we normally were but it didn’t impact the cost for us*.” [36].	
		Cost-offset CSA participants appreciated that they received FVs they would normally not be able to afford [22].	
Acceptance of SNAP benefits may facilitate participation	Enrollment	Focus group respondents were enthusiastic about using Supplemental Nutrition Assistance Program (SNAP) benefits to pay for the hypothetical CSA [29].	Accept SNAP benefits
		More than half of interviewees mentioned that payment options (e.g., SNAP) would facilitate hypothetical CSA participation (51%) [31].	
		More than half of interviewees (59%) were interested in a hypothetical CSA if they could pay with their SNAP benefits [17].	
	Engagement	Most cost-offset CSA participants used SNAP benefits to purchase their weekly farm shares (67%) [17].	
Close pick-up locations were preferred	Enrollment	Most interviewees were willing to travel 15 min or less (80%). Distance was particularly important to those who would walk (15%): ‘*I wouldn’t walk too far because I wouldn’t wanna carry it all back, so, you know, have a heavy load.*’ [34].	Offer convenient pick-up locations
	Satisfaction	CSA members from low-income households placed greater importance on a short distance to the CSA pick-up than their higher-income counterparts (4.0 vs. 3.7 on a 5-point scale) [30].	
		A total of 81% of cost-offset CSA participants reported challenges with picking up the CSA share, including distance [35].	
		Cost-offset CSA participants appreciated pick-up locations that were near their homes: “*It’s literally a mile from my house. It was very easy to just hop in the car, hop over there in the afternoon and then be done with it for the day.*” [36].	
Pick-up locations on existing travel routes were preferred	Enrollment	A total of 66% of interviewees wanted convenient pick-up location as a facilitator to hypothetical CSA participation: “*I mean, it just depends on the days that we would have to pick it up. Like if it was the same day that we go grocery shopping, that’d be fine ‘cause we’re driving anyways. But if it’s something that’s kind of inconvenient...*” [31].	
	Engagement	Pick-up rates were higher for cost-offset CSA participants whose children remained enrolled in Head Start (where the cost-offset CSA pick-up occurred) compared to those whose children withdrew and so they had to make a special trip for the FVs (81% vs. 57%) [17].	
	Satisfaction	Getting to the pick-up site was a substantial challenge for most cost-offset CSA participants. For many, share pick-up was ‘an extra errand’ because the location could not be integrated into normal travel routines [36].	
		Cost-offset CSA participants appreciated pick-up locations that were near routine daily activities like transporting children: “*It was very conveniently located for me because it was right by the school pickup. Preschool pickup on the day—so I could pick my kids up and on the way back we would go because it would be about 4:00 and we kinda made an activity of it*.” [33].	
Delivery of shares was desired	Enrollment	Many interviewees wanted their hypothetical CSA share delivered: “*Especially if it were delivered! I think for a lot of families that would take away huge barriers… ’cause I’m in such a time crunch and I even have a car, and I know lots of people that don’t have any way to get around. So, it wouldn’t matter how cheap things were, they couldn’t, they probably wouldn’t, be able to get out there and get [the share]*.” [31].	Provide delivery
	Satisfaction	When offered pick-up or delivery, most cost-offset CSA participants had their weekly share delivered (68%), but both groups strongly agreed or agreed that they received their shares without any difficulty (95%) [11].	
		Some cost-offset CSA participants requested delivery of shares: “*…one time* [farmer] *left our boxes on our doorstep and that was so amazing. I think the program deals with low-income people, that’s my understanding, people that are really super low-income, everything is exponentially more difficult for us.*” [33].	
Clear communication was valued	Satisfaction	Cost-offset CSA participants expressed frustration with the payment process, and some believed they had been double charged some weeks [22].	Prioritize clear communication
		Some cost-offset CSA participants found pick-up challenging because of poor organization or confusion regarding share contents or payments [36].	
		CSA members from households with low-income placed greater importance on ease of communication with CSA staff/farmers than their higher-income counterparts (3.9 vs. 3.5 on a 5-point scale) [30].	
		Cost-offset CSA participants described reminder calls/texts as important instrumental support [25].	
		Cost-offset CSA participants appreciated clear labelling at the pick-up location where FVs were self-selected [36].	
		Cost-offset CSA participants found newsletters and emails regarding share contents and pick-up timing to be useful [36].	

## Appendix A. Studies included in the scoping review and their characteristics

**Source**	**Location/Setting, Study Period, Intervention**	**Study Design**	**Sample(s)**	**Measures of Participation Assessed**
Abbott 2014, “Evaluation of the Food Bank of Delaware Community Supported Agriculture (CSA) Program” [22]	Wilmington, DE; 2013 **Intervention** - ~50% cost-offset CSA ($10/week full share; $5/week half share) - 18 week season - Supplemental Nutrition Assistance Program (SNAP) benefits accepted - Weekly newsletter with recipes and cooking demonstrations at pick-up	Qualitative analysis: Focus groups with cost-offset CSA participants	**Participant sample** (*n* = 208) Eligibility criteria: - Receive SNAP benefits **Focus group sample** *(n* = 10) Eligibility criteria: - English-speaking - Picked up the most shares	**Satisfaction** - Satisfaction with cost-offset CSA **Retention** - Interest in re-enrolling
Andreatta et al., 2008, “Lessons learned from advocating CSAs for low-income and food insecure households” [11]	Piedmont region of North Carolina; 2002–2003 **Intervention** - 100% cost-offset (free) CSA - Choice of share size from some farms - 20–30 week season - Weekly pick-up or delivery by volunteers - Printed information and recipes	Observational: Summary statistics and qualitative analysis of pre- and post-season interviews with participants	**Participant sample** (*n* = 22) Eligibility criteria: - Qualify for federal food assistance - Live within five miles of the pick-up location - Access to a ‘reasonably well-equipped’ kitchen	**Enrollment** - Cooking attitudes **Satisfaction** - Satisfaction with cost-offset CSA - Whether cost-offset CSA met expectations
Brehm and Eisenhauer 2008, “Motivations for Participating in Community-Supported Agriculture and Their Relationship with Community Attachment and Social Capital” [37]	Central Illinois and New Hampshire, 2006 **CSA Member survey** - Three CSAs in central Illinois - Four CSAs in New Hampshire	Observational: Cross-sectional analysis of participant surveys, compared across household incomes	**Participant sample** (*n* = 225) - Lower income, <$40,000 (26%) - Higher income, >$40,000 (74%)	**Enrollment** - Motivation for enrollment in CSA
Cotter et al., 2017, “Low-income adults’ perceptions of farmers’ markets and community supported agriculture programmes” [29]	Three affordable housing communities in Washington, DC; 2015–2016 **Hypothetical CSA**	Qualitative analysis: Focus groups	**Multi-lingual focus groups** (*n* = 28) Eligibility criteria: - Resident of affordable housing community - Speak English, Spanish or Amharic	**Enrollment** - Familiarity with CSA - Perceptions of hypothetical CSA
Galt et al., 2017, “What difference does income make for Community SupportedAgriculture (CSA) members in California: Comparinglower-income and higher-income households” [30]	CA statewide, 2014–2015 **CSA member survey** - 41 CSAs - Varied operational practices	Observational: Summary of cross-sectional survey data for low-income households (and compared to higher-income households)	**CSA member sample** (*n* = 1049) Eligibility criteria: - CSA members at the time of the survey - Low-income households (<$50,000/year, *n* = 129) - Higher-income households (≥$50,0000/year, *n* = 920)	**Enrollment** - Reasons for enrolling in CSA - Factors influencing the decision to enroll **Satisfaction** - Importance of CSA attributes
Garner et al., 2021, “Making community-supported agriculture accessible to low-income families: findings from the *Farm Fresh Foods for Healthy Kids (F3HK)* process evaluation” [23]	Rural and micropolitan communities in 4 U.S. states (NY, NC, VT, and WA); 2016–2017 **Intervention** - 50% cost-offset CSA (mean payment = $13/week) - Choice of share size from some farms - Weekly pick-up (mean = 21 weeks) - Choice of pick-up location from some farms - SNAP benefits accepted - FV preparation classes - Printed information and recipes - Kitchen tools	Observational: Cross-sectional analysis of - Applicant eligibility screening data - Enrollment data - Post-lesson surveys	Eligibility requirements: - Adult caregiver of a child aged 2–12 years - Household income ≤ 185% United States federal poverty level (FPL) - No CSA in at least 3 years - Able to speak and read English **Applicant sample** (*n* = 548) **Intervention sample** (*n* = 115)	**Enrollment** - Enrollment in cost-offset CSA **Engagement** - Pick-up rate - Used all or most of FVs in the share
Hanson et al., 2019, “Fruit and Vegetable Preferences and Practices may Hinder Participation in Community Supported Agriculture among Low-income Rural Families” [31]	Rural and micropolitan communities in 4 states (NY, NC, VT, and WA), 2015 **Hypothetical CSA**	Observational: Summary of recorded FV preferences Qualitative analysis: Interviews	**Adult Sample** (*n* = 41, ~10 from each state) Eligibility criteria: - Primary caregiver to a child aged 2–12 years - Household income ≤ 185% FPL - No CSA in the prior 3 years - Able to speak English **Child Sample** (*n* = 20, ~5 from each state) Eligibility criteria: - Child of adult interviewee - Aged 8–12 years	**Enrollment** - FV preferences - Knowledge, attitudes, and beliefs about participating in a hypothetical CSA
Hanson et al., 2019, “Knowledge, Attitudes, Beliefs and Behaviors Regarding Fruits and Vegetables among Cost-offset Community Supported Agriculture Applicants, Purchasers, and a Comparison Sample” [16]	VT statewide and comparison group from all New England states (MA, CT, RI, VT, NH, and ME); August 2017 **Cost-offset CSA member survey** - 50% cost-offset CSAs statewide (VT) - Varied operational practices	Observational: Cross-sectional comparison of online survey data from applicants and a non-applicant comparison group	Overall eligibility requirements: - Caregiver of a child aged 2–12 years - Household income ≤ 185% FPL - Able to speak and read English **Applicant sample** (*n* = 64) Applicant eligibility requirements: - Resided in VT - Applied to a cost-offset CSA program **Comparison sample** (*n* = 105) Comparison eligibility requirements: - Female - Resided in a New England state - No CSA in at least 3 years	**Enrollment** - Applied to a cost-offset CSA - Enrolled/purchased a cost-offset CSA
Hanson et al., 2022, “Participation in Cost-offset Community Supported Agriculture by Low-income Households in the U.S. is Associated with Community Characteristics and Operational Practices” [24]	Rural and micropolitan communities in 4 U.S. states (NY, NC, VT, and WA); 2016–2017 **Intervention** - 50% cost-offset CSA (mean payment = $13/week) - Choice of share size from some farms - Weekly pick-up (mean = 21 weeks) - Choice of pick-up locations from some farms - SNAP benefits accepted - FV preparation classes - Printed information and recipes - Kitchen tools	Observational: Examined differences in shares picked up (recorded in pick-up logs) by community and participant characteristics and CSA operational practices	**Participant sample** (*n* = 137) Eligibility requirements: - Adult caregiver of a child aged 2–12 years - Household income ≤ 185% FPL - No CSA in at least 3 years - Able to speak and read English - 8+ participants in community	**Engagement** - % of weeks shares were picked up
Hinrichs and Kremer 2002, “Social inclusion in a Midwest local food system project” [38]	Midwest, 1997–1998 **Intervention** - 100% or ‘partial’ cost-offset CSA - FV preparation classes	Observational: - Summary of post-intervention telephone surveys - Comparison of cost-offset CSA participants with full-price members	**CSA participant sample** (*n* = 41) - Cost-offset CSA (*n* = 13) - Full-price CSA (*n* = 28)	**Enrollment** - Enrollment - Reasons for enrolling **Satisfaction** - Perceptions of belonging to CSA
Hoffman et al., 2012, “Farm to Family: Increasing Access to Affordable Fruits and Vegetables Among Urban Head Start Families” [17]	Four Head Start centers in Boston, MA; 2010–2011 **Hypothetical CSA +** **Intervention** - 67% cost-offset CSA (mean payment = $5/week) - Weekly pick-up at Head Start - SNAP benefits accepted - Bilingual FV education	Observational: - Cross-sectional summary statistics from bilingual (English and Spanish) pre-intervention surveys - Cross-sectional analysis of post-intervention participant surveys	Overall eligibility criteria: - Caregiver of a child enrolled in Head Start - Able to speak and read English or Spanish **Pre-intervention Survey Sample** (*n* = 139) **Participant Sample** (*n* = 14) Participant eligibility criteria: - Enrolled in cost-offset CSA	**Enrollment** - Interest in a hypothetical CSA **Engagement** - Share pick-up rates **Satisfaction** - Satisfaction with cost-offset CSA Reasons for drop-out**Retention** - Interest in re-enrolling
Izumi et al., 2018, “Feasibility of Using a Community-Supported Agriculture Program to Increase Access to and Intake of Vegetables among Federally Qualified Health Center Patients” [25]	Federally qualified health center (FQHC), Portland, OR; 2015 **Intervention** - 75% cost-offset CSA (mean payment = $5/week) - Weekly pick-up at the clinic (23 weeks) - SNAP benefits accepted - Self-selected FVs - FV education and recipes - Reminders	Observational: Cross-sectional summary of pre- and post-intervention survey data Qualitative analysis: Post-intervention focus groups	**Participant survey sample** (*n* = 9) Eligibility criteria - FQHC clinic patient - Able to speak and read English or Spanish - No previous participation in CSA **Participant focus group sample** (*n* = 15)	**Enrollment** - Pre-intervention self-efficacy **Satisfaction** - Post-intervention satisfaction
Izumi et al., 2020, “CSA Partnerships for Health: outcome evaluation results from a subsidized community-supported agriculture program to connect safety-net clinic patients with farms to improve dietary behaviors, food security, and overall health” [26]	Federally qualified health center (FQHC), Portland, OR; 2017 **Intervention** - 78% cost-offset CSA (mean payment = $22/month) - Weekly pick-up at the clinic (23 weeks) - SNAP benefits accepted - Self-selected FVs - FV education and recipes - Reminders	Observational: Summary of pre- and post-intervention telephone and paper surveys	**Participant sample** (*n* = 48) Eligibility criteria - FQHC clinic patient - Able to speak and read English or Spanish	**Satisfaction** - Satisfaction with cost-offset CSA **Retention** - Interest in re-enrolling
Martin. et al., 2019, “Low-income mothers and the alternative food movement: An intersectional approach” [32]	3 North Carolina counties (1 urban and 2 rural); 2014–2015 **Hypothetical CSA**	Qualitative analysis: Semi-structured interviews about a hypothetical CSA	**Low-income sample** (*n* = 83) Eligibility criteria: - Mothers/grandmothers of young children - Low income (not defined) - English speaking - No previous participation in CSA	**Enrollment** - Familiarity with CSA - Perceptions of hypothetical CSA
McGuirt et al., 2018, “A Modified Choice Experiment to Examine Willingness to Participate in a Community Supported Agriculture (CSA) Program among Low-income Parents” [34]	Rural and micropolitan communities in 4 U.S. states (NY, NC, VT, and WA); 2015 **Hypothetical CSA**	Observational: Summary of a modified choice experiment about a hypothetical CSA (travel distance, share size, share frequency, and price) conducted during interviews Qualitative analysis of interview comments	**Sample** (*n* = 42, ~10 from each state) Eligibility criteria: - Primary caregiver to a child aged 2–12 years - Household income ≤ 185% FPL - No CSA in prior 3 years - Able to speak and read English	**Enrollment** - Attitudes toward hypothetical CSA
McGuirt et al., 2019, “A mixed-methods examination of the geospatial and sociodemographic context of a direct-to-consumer food system innovation” [33]	Rural and micropolitan communities in 4 U.S. states (NY, NC, VT, and WA); 2016–2017 **Intervention** - 50% cost-offset CSA (mean payment =$13/week) - Choice of share sizes from some farms - Weekly pick-up (mean = 21 weeks) - Choice of pick-up locations from some farms - SNAP benefits accepted - FV preparation classes - Printed information and recipes - Kitchen tools	Observational: Geospatial analysis of participants’ homes, CSA farms, and pick-up locations Qualitative analysis: Focus groups with participants	**Geospatial data** - Participants’ homes (*n* = 92) - CSA farms (*n* = 12) - Pick-up locations (*n* = 16) **Participant sample** (*n* = 53) Eligibility criteria: - Primary caregiver to child 2–12*y* - Household income ≤ 185% FPL - No CSA in prior 3 years - Able to speak and read English	**Satisfaction** - Satisfaction with pick-up location - Socio-demographic context of pick-up location
Quandt et al., 2013, “Feasibility of using a Community-Supported Agriculture Program to improve fruit and vegetable inventories and consumption in an underresourced urban community” [35]	Forsyth County, NC; 2012 **Intervention** - 100% cost-offset CSA (free) - Weekly pick-up - FV preparation classes and recipes	Observational: Descriptive analysis of post-intervention telephone interviews	**Participant sample** (*n* = 25) Eligibility criteria: - Clients of a community action agency - Female - Head of household with 1+ children - Able to speak English	**Satisfaction** - Satisfaction with cost-offset CSA **Retention** - Interest in re-enrolling - Willingness-to-pay for future cost-offset CSA
White et al., 2018, “The perceived influence of cost-offset community-supported agriculture on food access among low-income families” [36]	Rural and micropolitan communities in 4 U.S. states (NY, NC, VT, and WA); 2016–2017 **Intervention** - 50% cost-offset CSA (mean payment =$13/week) - Choice of share sizes from some farms - Weekly pick-up (mean = 21 weeks) - Choice of pick-up locations from some farms - SNAP benefits accepted - FV preparation classes - Printed information and recipes - Kitchen tools	Qualitative analysis: Post-intervention focus groups	**Participant sample** (*n* = 53) Eligibility criteria: - Primary caregiver to a child aged 2–12 years - Household income ≤ 185% FPL - No CSA in the prior 3 years - Able to speak and read English	**Satisfaction** - Satisfaction with cost-offset CSA

## References

[1] Slavin J.L., Lloyd B. (2012). Health benefits of fruits and vegetables. Adv. Nutr..

[2] Snetselaar L.G., de Jesus J.M., DeSilva D.M., Stoody E.E. (2021). Dietary guidelines for Americans, 2020–2025: Understanding the scientific process, guidelines, and key recommendations. Nutr. Today.

[3] Bruening M., MacLehose R., Loth K., Story M. (2012). Neumark-Sztainer D: Feeding a family in a recession: Food insecurity among Minnesota parents. Am. J. Public Health.

[4] Champagne C.M., Casey P.H., Connell C.L., Stuff J.E., Gossett J.M., Harsha D.W., McCabe-Sellers B., Robbins J.M., Simpson P.M., Weber J.L. (2007). Poverty and food intake in rural America: Diet quality is lower in food insecure adults in the Mississippi Delta. J. Am. Diet. Assoc..

[5] Cristofar S.P., Basiotis P.P. (1992). Dietary intakes and selected characteristics of women ages 19–50 years and their children ages 1–5 years by reported perception of food sufficiency. J. Nutr. Educ..

[6] Dixon L.B., Winkleby M.A., Radimer K.L. (2001). Dietary intakes and serum nutrients differ between adults from food-insufficient and food-sufficient families: Third National Health and Nutrition Examination Survey, 1988–1994. J. Nutr..

[7] Grimm K.A., Foltz J.L., Blanck H.M., Scanlon K.S. (2012). Household income disparities in fruit and vegetable consumption by state and territory: Results of the 2009 Behavioral Risk Factor Surveillance System. J. Acad. Nutr. Diet..

[8] Kendall A., Olson C.M., Frongillo E.A. (1996). Relationship of hunger and food insecurity to food availability and consumption. J. Am. Diet. Assoc..

[9] Lee S.H. (2022). Adults meeting fruit and vegetable intake recommendations—United States, 2019. MMWR Morb. Mortal. Wkly. Rep..

[10] Bonfert B. (2022). ‘What we’d like is a CSA in every town.’Scaling community supported agriculture across the UK. J. Rural Stud..

[11] Andreatta S., Rhyne M., Dery N. (2008). Lessons learned from advocating CSAs for low-income and food insecure households. J. Rural Soc. Sci. (Former. South. Rural Sociol.).

[12] Guthman J., Morris A.W., Allen P. (2006). Squaring farm security and food security in two types of alternative food institutions. Rural Sociol..

[13] Pitts S.B.J., Volpe L.C., Sitaker M., Belarmino E.H., Sealey A., Wang W., Becot F., McGuirt J.T., Ammerman A.S., Hanson K.L. (2022). Offsetting the cost of community-supported agriculture (CSA) for low-income families: Perceptions and experiences of CSA farmers and members. Renew. Agric. Food Syst..

[14] Agboola F. (2017). Implications of Community Supported Agriculture as Alternative Food Networks.

[15] Berkowitz S.A., O’Neill J., Sayer E., Shahid N.N., Petrie M., Schouboe S., Saraceno M., Bellin R. (2019). Health center–based community-supported agriculture: An RCT. Am. J. Prev. Med..

[16] Hanson K.L., Volpe L.C., Kolodinsky J., Hwang G., Wang W., Jilcott Pitts S.B., Sitaker M., Ammerman A.S., Seguin R.A. (2019). Knowledge, attitudes, beliefs and behaviors regarding fruits and vegetables among cost-offset community-supported agriculture (CSA) applicants, purchasers, and a comparison sample. Nutrients.

[17] Hoffman J.A., Agrawal T., Wirth C., Watts C., Adeduntan G., Myles L., Castaneda-Sceppa C. (2012). Farm to family: Increasing access to affordable fruits and vegetables among urban head start families. J. Hunger Environ. Nutr..

[18] Johnson D.B., Beaudoin S.L., Beresford S.A., LoGerfo J.P. (2004). Increasing fruit and vegetable intake in homebound elders: The Seattle Farmers’ Market nutrition pilot program. Prev. Chronic Dis..

[19] Kato Y., McKinney L. (2015). Bringing food desert residents to an alternative food market: A semi-experimental study of impediments to food access. Agric. Hum. Values.

[20] Seguin-Fowler R.A., Hanson K.L., Jilcott Pitts S.B., Kolodinsky J., Sitaker M., Ammerman A.S., Marshall G.A., Belarmino E.H., Garner J.A., Wang W. (2021). Community supported agriculture plus nutrition education improves skills, self-efficacy, and eating behaviors among low-income caregivers but not their children: A randomized controlled trial. Int. J. Behav. Nutr. Phys. Act..

[21] Wilkins J.L., Farrell T.J., Rangarajan A. (2015). Linking vegetable preferences, health and local food systems through community-supported agriculture. Public Health Nutr..

[22] Abbott C. (2014). Evaluation of the Food Bank of Delaware Community Supported Agriculture Program.

[23] Garner J.A., Jilcott Pitts S.B., Hanson K.L., Ammerman A.S., Kolodinsky J., Sitaker M.H., Seguin-Fowler R.A. (2021). Making community-supported agriculture accessible to low-income families: Findings from the Farm Fresh Foods for Healthy Kids process evaluation. Transl. Behav. Med..

[24] Hanson K.L., Xu L., Marshall G.A., Sitaker M., Jilcott Pitts S.B., Kolodinsky J., Bennett A., Carriker S., Smith D., Ammerman A.S. (2022). Participation in Cost-offset Community Supported Agriculture by Low-income Households in the U.S. is Associated with Community Characteristics and Operational Practices. Public Health Nutr..

[25] Izumi B.T., Higgins C.E., Baron A., Ness S.J., Allan B., Barth E.T., Smith T.M., Pranian K., Frank B. (2018). Feasibility of using a community-supported agriculture program to increase access to and intake of vegetables among federally qualified health center patients. J. Nutr. Educ. Behav..

[26] Izumi B.T., Martin A., Garvin T., Higgins Tejera C., Ness S., Pranian K., Lubowicki L. (2020). CSA Partnerships for Health: Outcome evaluation results from a subsidized community-supported agriculture program to connect safety-net clinic patients with farms to improve dietary behaviors, food security, and overall health. Transl. Behav. Med..

[27] Mak S., Thomas A. (2022). An Introduction to Scoping Reviews. J. Grad. Med. Educ..

[28] Tricco A.C., Lillie E., Zarin W., O’Brien K.K., Colquhoun H., Levac D., Moher D., Peters M.D., Horsley T., Weeks L. (2018). PRISMA extension for scoping reviews (PRISMA-ScR): Checklist and explanation. Ann. Intern. Med..

[29] Cotter E.W., Teixeira C., Bontrager A., Horton K., Soriano D. (2017). Low-income adults’ perceptions of farmers’ markets and community-supported agriculture programmes. Public Health Nutr..

[30] Galt R.E., Bradley K., Christensen L., Fake C., Munden-Dixon K., Simpson N., Surls R., Van Soelen Kim J. (2017). What difference does income make for Community Supported Agriculture (CSA) members in California? Comparing lower-income and higher-income households. Agric. Hum. Values.

[31] Hanson K.L., Garner J., Connor L.M., Pitts S.B.J., McGuirt J., Harris R., Kolodinsky J., Wang W., Sitaker M., Ammerman A. (2019). Fruit and vegetable preferences and practices may hinder participation in community-supported agriculture among low-income rural families. J. Nutr. Educ. Behav..

[32] Martin B., Mycek M.K., Elliott S., Bowen S. (2019). Low-income mothers and the alternative food movement: An intersectional approach. Feminist Food Studies: Intersectional Perspectives.

[33] McGuirt J., Sitaker M., Pitts S.J., Ammerman A., Kolodinsky J., Seguin-Fowler R. (2019). A mixed-methods examination of the geospatial and sociodemographic context of a direct-to-consumer food system innovation. J. Agric. Food Syst. Community Dev..

[34] McGuirt J.T., Pitts S.B.J., Hanson K.L., DeMarco M., Seguin R.A., Kolodinsky J., Becot F., Ammerman A.S. (2018). A modified choice experiment to examine willingness to participate in a Community Supported Agriculture (CSA) program among low-income parents. Renew. Agric. Food Syst..

[35] Quandt S.A., Dupuis J., Fish C., D’Agostino R.B. (2013). Feasibility of using a community-supported agriculture program to improve fruit and vegetable inventories and consumption in an underresourced urban community. Prev. Chronic Dis..

[36] White M.J., Jilcott Pitts S.B., McGuirt J.T., Hanson K.L., Morgan E.H., Kolodinsky J., Wang W., Sitaker M., Ammerman A.S., Seguin R.A. (2018). The perceived influence of cost-offset community-supported agriculture on food access among low-income families. Public Health Nutr..

[37] Brehm J.M., Eisenhauer B.W. (2008). Motivations for participating in community-supported agriculture and their relationship with community attachment and social capital. J. Rural Soc. Sci..

[38] Hinrichs C., Kremer K.S. (2002). Social inclusion in a Midwest local food system project. J. Poverty.

[39] Flagg L.A., Sen B., Kilgore M., Locher J.L. (2014). The influence of gender, age, education and household size on meal preparation and food shopping responsibilities. Public Health Nutr..

[40] Larson N.I., Story M., Eisenberg M.E., Neumark-Sztainer D. (2006). Food Preparation and Purchasing Roles among Adolescents: Associations with Sociodemographic Characteristics and Diet Quality. J. Am. Diet. Assoc..

[41] Mills S., White M., Brown H., Wrieden W., Kwasnicka D., Halligan J., Robalino S., Adams J. (2017). Health and social determinants and outcomes of home cooking: A systematic review of observational studies. Appetite.

[42] Villar C. (2017). Engaging Past and Future on a Community Supported Agriculture Farm.

[43] Cicatiello C., Franco S., Pancino B., Blasi E., Falasconi L. (2017). The dark side of retail food waste: Evidences from in-store data. Resour. Conserv. Recycl..

[44] Cruwys T., Beyelander K.E., Hermans R.C.J. (2015). Social modeling of eating: A review of when and why social influence affects food intake and choice. Appetite.

[45] Ventura A.K., Worobey J. (2013). Early influences on the development of food preferences. Curr. Biol..

[46] Prial D. (2019). Community Supported Agriculture. ATTRA Sustainable Agriculture.

[47] Fox D. (2017). CSAs in the Capital Region: How they work. Times Union. https://www.timesunion.com/tuplus-features/article/CSAs-in-the-Capital-Region-How-they-work-11004701.php.

[48] Sitaker M., McCall M., Wang W.W., Vaccaro M., Kolodinsky J.M., Ammerman A., Pitts S.J., Hanson K., Smith D.K., Seguin-Fowler R.A. (2021). Models for cost-offset community supported agriculture (CO-CSA) programs. J. Agric. Food Syst. Community Dev..

[49] Woods T., Ernst M., Tropp D. (2017). Community Supported Agriculture: New Models for Changing Markets.

[50] SNAP Participation Rates by State, All Eligible People (FY 2019). https://www.fns.usda.gov/usamap.

[51] Bruch M.L., Ernst M.D. (2010). A Farmer’s Guide to Marketing through Community Supported Agriculture (CSAs).

[52] Farmers Markets Accepting SNAP Benefits. https://www.fns.usda.gov/snap/farmers-markets-accepting-snap-benefits.

[53] De Graaf J. (2003). Take back Your Time: Fighting Overwork and Time Poverty in America.

[54] Shrider E.A., Kollar M., Chen F., Semega J. (2021). Income and Poverty in the United States: 2020.

[55] Lubbers M.J., García H.V., Castaño P.E., Molina J.L., Casellas A., Rebollo J.G. (2020). Relationships Stretched Thin: Social Support Mobilization in Poverty. Ann. Am. Acad. Political Soc. Sci..

[56] Radey M., McWey L.M. (2019). Informal Networks of Low-Income Mothers: Support, Burden, and Change. J. Marriage Fam..

[57] Corporation L.S. (2022). The Justice Gap: The Unmet Civil Legal Needs of Low Income Americans.

[58] How to Create a Crop Plan for Your CSA in 5 Steps. https://howtostartanllc.com/csa/how-to-create-crop-plan.

[59] Sitaker M., McCall M., Belarmino E., Wang W., Kolodinsky J., Becot F., McGuirt J., Ammerman A., Pitts S.J., Seguin-Fowler R. (2020). Balancing social values with economic realities: Farmer experience with a cost-offset CSA. J. Agric. Food Syst. Community Dev..

[60] Verfuerth C., Sanderson Bellamy A., Adlerova B., Dutton A. (2023). Building relationships back into the food system: Addressing food insecurity and food well-being. Front. Sustain. Food Syst..

[61] Janssen B. (2010). Local Food, Local Engagement: Community-Supported Agriculture in Eastern Iowa. Cult. Agric..

